# The meaning of significance in data testing

**DOI:** 10.3389/fpsyg.2015.01293

**Published:** 2015-08-27

**Authors:** Jose D. Perezgonzalez

**Affiliations:** Business School, Massey UniversityPalmerston North, New Zealand

**Keywords:** statistical significance, practical significance, statistical misinterpretations, research pedagogy, epistemology in psychology

Recent developments in psychology (e.g., Nuzzo, 2014; Trafimow, 2014; Woolston, 2015a) are showing apparently reasonable but inherently flawed positions against data testing techniques (often called hypothesis testing techniques, even when they do not test hypotheses but assume them true for testing purposes). These positions are such as banning testing explicitly and most inferential statistics implicitly (Trafimow and Marks, 2015, for *Basic and Applied Social Psychology*—but see Woolston, 2015a, expanded in http://www.nature.com/news/psychology-journal-bans-p-values-1.17001), recommending substituting confidence intervals for null hypothesis significant testing (NHST) explicitly and for all other data testing implicitly (Cumming, 2014, for *Psychological Science*—but see Perezgonzalez, 2015a; Savalei and Dunn, 2015), and recommending research preregistration as a solution to the low publication of non-significant results (e.g., Woolston, 2015b). In reading Woolston's articles, readers' comments to such articles, and the related literature, it appears that philosophical misinterpretations of old, already discussed by, for example, Meehl (1997), Nickerson (2000), Kline (2004), and Goodman (2008), are not getting through and still need to be re-addressed today. I believe that a chief source of misinterpretations is the current NHST framework, an incompatible mishmash between the testing theories of Fisher and of Neyman-Pearson (Gigerenzer, 2004). The resulting misinterpretations have both a statistical and a theoretical background. Statistical misinterpretations of *p*-values have been addressed elsewhere (Perezgonzalez, 2015c), thus I reserve this article for resolving theoretical misinterpretations regarding statistical significance.

The main confusions regarding statistical significance can be summarized in the following seven points (e.g. Kline, 2004): (1) significance implies an important, real effect size; (2) no significance implies a trivial effect size; (3) significance disproves the tested hypothesis; (4) significance proves the alternative hypothesis; (5) significance exonerates the methodology used; (6) no significance is explainable by bad methodology; and (7) no significance in a follow up study means a replication failure. These seven points can be discussed according to two concerns: the meaning of significance itself, and the meaning, or role, of testing.

In this article I will avoid NHST and, instead, refer to either Fisher's or Neyman-Pearson's approaches, when appropriate. I will also avoid their conceptual mix-up by using different concepts, those which seem most coherent under each approach. Thus, Fisher's seeks significant results, tests data on a null hypothesis (H_0_) and uses levels of significance (sig) to ascertain the probability of the data under H_0_ (Figure 1A). Neyman-Pearson's seeks to make a decision, tests data on a main hypothesis (H_M_) and decides in favor of an alternative hypothesis (H_A_) according to a cut-off calculated a priori based on sample size (N), Type I error probability (α), effect size (MES) and power (1-β), the latter two provided by H_A_ (Figure 1B).

**Figure 1:**
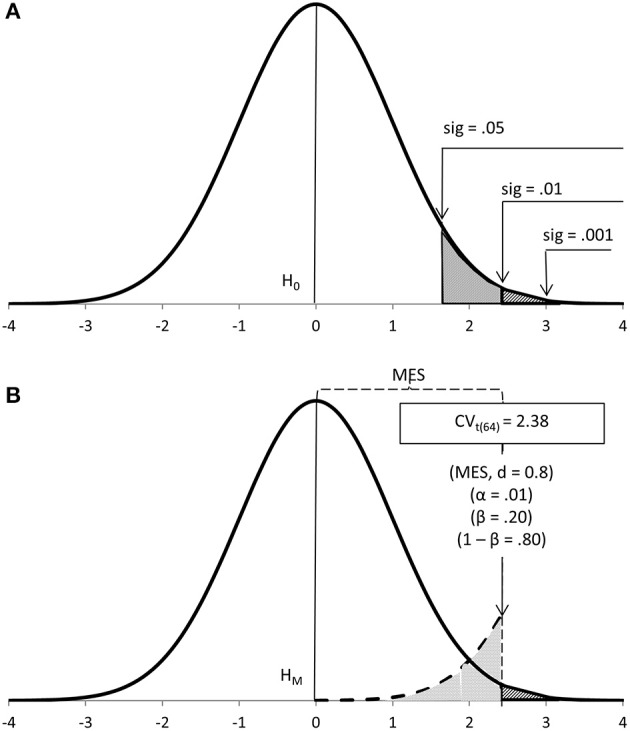
**Fisher's (A) and Neyman-Pearson's (B) approaches to data testing mapped onto a ***t***-test distribution with 64 degrees of freedom**.

## The meaning of significance

Misinterpretations (1) and (2) are due to confusing statistical significance, theoretical or practical significance, and effect sizes. The latter are a property of populations, may vary from large to small, can be put into a number, and can be calculated with the appropriate formula (Cohen, 1988). Practical significance is a subjective assessment of the importance of such effect (they can be considered as two sides of the same coin).

As per statistical significance, because testing is done on samples, it is the equivalent population effects in the corresponding sampling distributions which are of relevance, to be found in the tail (or tails) of such distributions. When using these techniques, therefore, important effects become extreme results—i.e., results with low *p*-values—under the tested hypotheses.

To help with inferences, a cut-off is used to partition the corresponding sampling distribution between extreme and not-extreme-enough results. This is, of course, a pragmatic choice, but it implies that the meaning of significance ultimately depends on where such cut-off falls. This cut-off also partitions the effect size between important and unimportant effects for testing purposes.

Under Neyman-Pearson's approach (e.g., 1933; Figure 1B), the mathematically-set cut-off partitions the effect size a priori (the minimum effect size, MES, is the value of the population effect size at such point; Perezgonzalez, 2015b). Statistical significance, thus, has no inherent meaning under this approach other than to identify extreme results beyond the set cut-off. Because the sample size is controlled, mainly to ensure power, such extreme results are not only more probable under H_A_ but also reflect important population effects.

Under Fisher's approach (e.g., 1954, 1960; Figure 1A), either experience-driven or conventional cut-offs help flag noteworthy results—these are properly significant, as in “notable,” “worthy of attention”—whose primary value is in their role as evidence for rejecting H_0_. Because there is no inherent control of sample size, a large sample may be used if it leads to the rejection of H_0_ more readily—thus, a significant result is technically important. Whether it is really important, however, we cannot know (we ought to wait and calculate the effect size a posteriori), but we may assume an unknown MES with boundaries at the appropriate level of significance. Posterior calculations normally shows that when the sample is small, significant results reflect large effect sizes; as the sample grows larger, the resulting effect sizes may shrink into triviality.

Curiously, then, misinterpretations (1) and (2) are only possible under Fisher's approach depending ultimately on the size of the sample used. With small samples—which is the paradigm that Fisher developed—significant results normally do reflect important effects—thus, (1) is typically not a misinterpretation—however some of the non-significant results may also reflect sizeable effects—thus, misinterpretation (2) is still possible. The opposite occurs with larger samples: Effect sizes may be of any size, including negligible ones, and still turn out statistically significant—thus, misinterpretation (1) is plausible—while non-significant results will often be negligible—thus, misinterpretation (2) is not so, but a correct interpretation.

Under Neyman-Pearson's approach, on the other hand, effect sizes are those of populations, known (or fixed) before conducting the research. These effect sizes can, of course, be set differently by different researchers, yet such decision has a technical consequence on the test thereof: It makes a posteriori interpretations of effect sizes meaningless. Thus, an extreme result—accepted under H_A_—is always important because the researcher decided so when setting the test; a not-so-extreme result—accepted under H_M_—is always trivial for similar reasons; therefore, as far as any particular test result is concerned, (1) and (2) cannot be considered misinterpretations proper under this approach.

## The meaning of data testing

The remaning misinterpretations have to do with confusing research substance and data testing technicality. Meehl (1997) provided clear admonition about the substantive aspects of theory appraisal. He set down a conceptual formula for correlating a set of observations with a theory and related components. His formula includes not only the theory under test—from which the statistical hypothesis supposedly flaws—but also auxiliary theories, the everything-else-being-equal assumption (*ceteris paribus*), and reporting quality—all of which address misinterpretations (3) and (4)—as well as methodological quality—which addresses misinterpretations (5) and (6). Thus, the observation of a significant or extreme result is, at most, able to falsify only the conjunction of elements in the formula instead of the theory under test—i.e., either the theory is false, or the auxiliary theories are false, or the *ceteris paribus* clause is false, or the particulars reported are wrong, or the methodology is flawed. Furthermore, Meehl argues, following the Popperian dictum a theory cannot be proved, so a non-significant or not-extreme-enough result cannot be used for such purpose, either.

Meehl may have slipped on the technicality of testing, though, still confusing a substantive hypothesis—albeit a very specific one—with a statistical hypothesis. Technically speaking, a statistical hypothesis (H_0_, H_M_, H_A_) provides the appropriate frequency distribution for testing research data and, thus, needs to be (technically) true. Therefore, these hypotheses cannot be either proved or disproved—i.e., disproving a statistical hypothesis invalidates both the test and the results used to disprove it! From this follows that the gap between the statistical hypothesis and the related substantive hypothesis that supposedly flaws from the theory under appraisal cannot be closed statistically but only epistemologically (Perezgonzalez, 2015b).

Therefore, misinterpretations (3) and (4) have conflating technical and substantive causes. Meehl's (1997) formula resolves the substantive aspect, while a technical argument can also be advanced as a solution: Statistical hypotheses need to be (assumed) true and, thus, can be neither proved nor disproved by the research data.

As for misinterpretations (5) and (6), about methodology, these too are resolved by Meehl's formula. Methodological quality is a necessary element for theory appraisal, yet also an independent element in the formula; thus, we may observe a particular research result independently of the quality of the methods used. This is something which is reasonable and may need no further discussion, yet it is also something which tends to appear divorced from the research process in psychological reporting. Indeed, psychological articles tend to address research limitations only at the end, in the discussion and conclusion section (see, for example, American Psychological Association's style recommendations, 2010), something which reads more as an act of contrition than as reassurance that those limitations have been taken into account in the research.

Finally, a technical point can also be advanced for resolving the replication misinterpretation (7). Depending on the approach used, replication necessitates either of a cumulative meta-analysis (Fisher's approach; Braver et al., 2014) or of a count of the number of replications carried out (Neyman-Pearson's approach; Perezgonzalez, 2015a,d). A single replication may suffix the former, yet it is the significance of the meta-analysis, not of the individual studies, that counts. As for the latter, one would expect a minimum number of replications (i.e., four) in order to ascertain the power of the study (i.e., a minimum of four successful studies out of five for ascertaining 80% power); a single replication is, thus, not enough. Therefore, the significance or extremeness of a single replication cannot be considered enough ground for either supporting or contradicting a previous study.

## Corollary

Late developments in the editorial policies for the journals *Basic and Applied Social Psychology*, and *Psychological Science* aim to improve the quality of the papers submitted for publication (similar attempts have already been attempted in the past—e.g., Loftus, 1993; Kendall, 1997—with rather limited success—e.g., Finch et al., 2004; Fidler et al., 2005). They do so by banning or strongly discouraging the use of inferential tools, more specifically data testing procedures. There are important theoretical and philosophical reasons for supporting the banning of NHST (e.g., Nickerson, 2000), but these do not necessarily extend to either Fisher's or Neyman-Pearson's procedures or to the remaining of the inferential toolbox. The main problem seems to lie with misinterpretations borne out of NHST and the way statistics is taught. *P*-values are often misinterpreted as providing information they do not—something that may be resolved by simply substituting frequency-based heuristics for the probabilistic heuristics currently used (e.g., Perezgonzalez, 2015c). On the other hand, statistical significance is often misinterpreted as practical importance. Substantive arguments about theory appraisal can resolve some of the misinterpretations, although this requires some reading about epistemology (e.g., Meehl, 1997). Furthermore, technical arguments can also be advanced to resolve other misinterpretations. Many of these confusions could be easily captured and prevented at pedagogical levels, thus highlighting the important role of doing so when teaching statistics. The risk of not doing so is transferred forward to the rest of psychology, which may suffer when misunderstood testing procedures and other inferential tools are discouraged or banned outright for the purpose of, paradoxically, improving psychological science.

### Conflict of interest statement

The author declares that the research was conducted in the absence of any commercial or financial relationships that could be construed as a potential conflict of interest.
